# A group-housing system for an artificial shark uterus

**DOI:** 10.1016/j.mex.2026.103905

**Published:** 2026-04-09

**Authors:** Taketeru Tomita, Atsushi Kaneko, Tomoya Takeda, Keiichi Sato

**Affiliations:** a Okinawa Churashima Research Institute, Okinawa Churashima Foundation, Motobu, Japan; b Okinawa Churaumi Aquarium, Okinawa Churashima Foundation, Motobu, Japan

**Keywords:** Artificial womb, Artificial uterine fluid, Conservation breeding, Elasmobranchs, Embryo, Incubation

## Abstract

We developed an artificial shark uterus system that enables the simultaneous maintenance of multiple embryos within a single container. In previous designs, group housing of late-stage embryos was unsuccessful because the activation of one embryo stimulated others, triggering collective hyperactivity. This led to severe skin abrasions due to repeated contact with the rubber mesh covering the container opening.

The key modifications and functional principle of the new system are as follows:

• The rubber mesh was eliminated and replaced with an acrylic cylinder that provides vertical space above the incubation container.
• This vertical space allows activated embryos to swim upward, reducing physical contact between active and inactive individuals.

This method increases incubation density and broadens the applicability of artificial uterine technology to shark species with high fecundity.

## Graphical abstract

**Figure:**
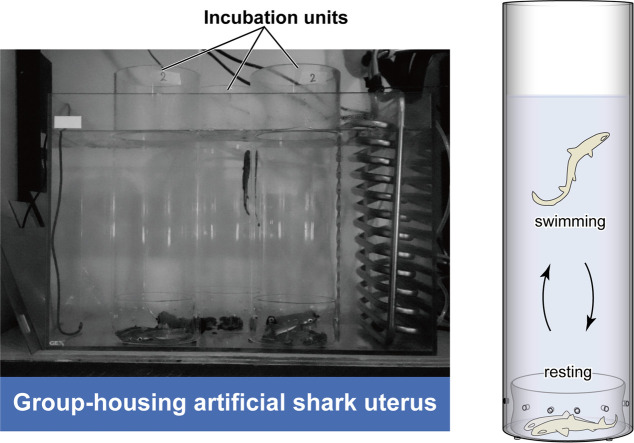

**Specifications table**

**Table:** 

Subject area	Veterinary Science and Veterinary Medicine
**More specific subject area**	Artificial uterine science
**Name of your method**	Group-housing artificial shark uterus
**Name and reference of original method**	Not applicable
**Resource availability**	Not applicable

## Background

An extrauterine life support system, also referred to as an artificial uterus, is a medical technology that sustains prematurely-born embryos or fetuses *ex utero*. Initially developed for mammals, this technology was later extended to viviparous sharks for veterinary and conservation breeding purposes [1]. Since 2017, we have constructed artificial uterus systems and successfully maintained embryos of the deepwater shark *Etmopterus schmidti* for up to one year until artificial delivery [[2], [3], [4], [5]]. These systems utilize an artificial uterine fluid (AUF), a urea-containing solution with osmotic pressure and salinity nearly equivalent to those of shark blood plasma. This fluid was designed to reduce osmoregulatory stress in embryos, and its use extended the survival period of the shark embryo from less than one week to several months [2], achieving a survival rate of approximately 90 % [5].

Despite these advances, a major limitation of the current system is its low embryo capacity. This constraint becomes critical when applying the system to shark species with high fecundity (e.g., >30 embryos per pregnancy). In the current design, each embryo is housed individually in a small glass or plastic container, which is placed inside a larger tank filled with AUF [2,5]. The top of each container is covered with a silicone rubber mesh that allows fluid exchange with the external environment. The concept of an individual-housing system was derived from a study of Gilbert [6], who found that the restriction of embryo movement prevented abrasion of the fragile embryonic skin, increasing the survival rate. However, it requires substantial space per individual, thereby limiting the number of embryos that can be maintained simultaneously.

One possible solution to this limitation would be to incubate multiple embryos within a single container. This group housing is more similar to that of the natural uterus, which includes multiple embryos in the same chamber. However, in the current system, such an approach was unsuccessful. Late-stage embryos (stage 33) are highly sensitive to physical stimuli. Once activated, they may swim continuously against the container wall and rubber mesh for extended periods, resulting in severe skin abrasions. In a group-housing method, activation of one individual may propagate among co-housed embryos, elevating the overall activity levels. This collective hyperactivity may also increase the risk of hypoxia due to a rapid elevation of oxygen consumption within the container. Accordingly, during incubation trials conducted from 2022 to 2023, we initially maintained mid-term embryos under group housing (up to three embryos per container) but ultimately separated them into individual containers by stage 33 when the snout skin abrasions became prominent [5].

Here, we describe a modified artificial uterus system that enables higher-density group housing while minimizing activation cascades and associated oxygen depletion.

## Method details

### System design

**Fig. 1:**
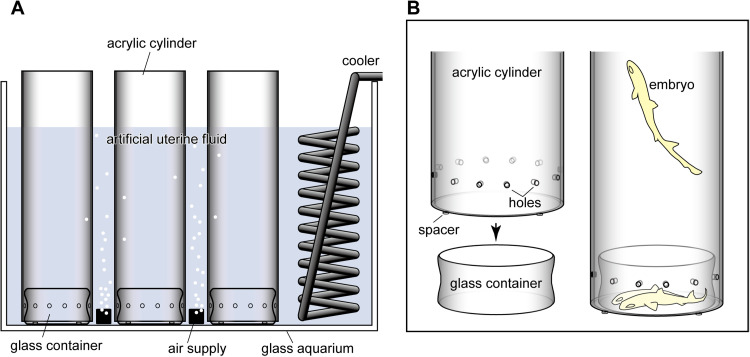
Group housing system for artificial shark uterus. **A**. Overall system. Multiple acrylic cylinder-glass container units are arranged in a larger aquarium including artificial uterine fluid. **B**. Close-up view of the lower part of the incubation unit.

The overall system is shown in Fig. 1. Multiple open-ended acrylic cylinders (internal diameter: 13.7 cm; height: 44.0 cm; wall thickness: 6 mm) were positioned vertically within a 70-L rectangular aquarium filled with AUF (see Tomita et al [2,3] for construction details). The water level was maintained below the upper rim of each cylinder.

For continuous fluid exchange, twelve holes (5.0 mm in diameter) were created in the lateral wall of each cylinder, positioned 4 cm above the base. In addition, three silicone spacers (5.0 mm in width and 2.0 mm in height) were attached to the lower rim of each cylinder to elevate it slightly above the aquarium floor, thereby enhancing fluid circulation.

Within each acrylic cylinder, a cylindrical glass incubation container (internal diameter: 13 cm; internal height: 6.5 cm) was placed directly on the aquarium bottom, and embryos were housed in this container. Aeration was provided outside the acrylic cylinders near the bottom of the aquarium, ensuring indirect water movement without generating strong currents within the incubation container. Water temperature was maintained at 12 °C using an in-tank cooling unit. The system was placed in a dark room with dim red illumination.

### Functional principle

This system was designed to spatially separate active and inactive embryos within a group-housing environment. When inactive, embryos rest on the bottom of the glass container. When some of the embryos are activated, they swim upward into the space within the acrylic cylinder. This vertical displacement reduces physical contact between active and inactive individuals, thereby limiting mechanical stimulation and minimizing the interaction stress and propagation of activation.

## Method validation

### Acquisition of shark embryos

Four pregnant females of *Etmopterus schmidti* were obtained from local fishermen on January 24, 2026. They were caught by hook and line fishing at a depth of approximately 500 m off the main island of Okinawa, Japan. The females died during the onboard holding or during the 6-h return trip and were donated to the Okinawa Churaumi Aquarium (Okinawa, Japan) on the day of capture.

A total of 25 embryos were obtained through dissection. All embryos possessed small external yolk sacs (1–5 mm) and lacked external gill filaments. According to the developmental staging criteria of Ballard et al [7], all embryos belonged to stage 33. Immediately after recovery, embryos were maintained individually in 500-mL plastic bottles containing AUF at 12 °C and were transferred to the experimental system on the day of the system setup.

### Experimental design

**Fig. 2:**
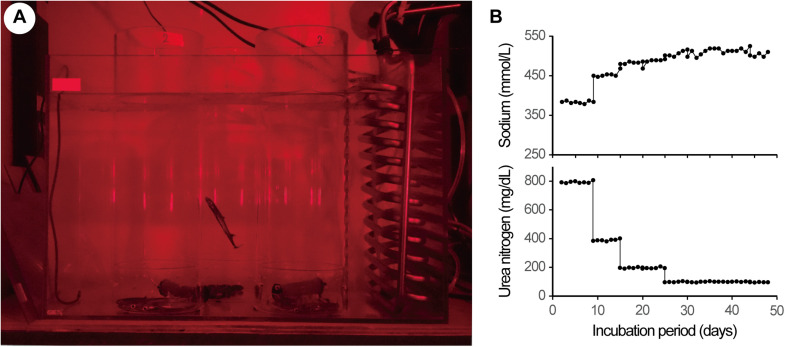
Application of the new system to the deepwater shark *Etmopterus schmidti* embryos. **A**. Three acrylic cylinder-container units were arranged in the 70-L aquarium (Aquarium 1). Each unit included 4 embryos. **B**. Change in the concentrations of sodium (top), and urea (bottom) of the incubation fluid.

Embryos were distributed among six cylinder-container incubation units, with 3–6 embryos per unit (Fig. 2A). Three units were installed in each of two 70-L aquariums (Aquarium 1 and Aquarium 2), each containing 50 L of the incubation fluid maintained at 12 °C. Embryonic condition was assessed twice daily (09:00 and 17:00) by observing respiratory movements (gill slits and spiracular valve motion) and behavioral responses to light stimuli (body undulation and posture changes).

In previous studies [[3], [4], [5]], embryos were acclimated gradually from AUF to seawater in a stepwise manner. In the present study, given the advanced developmental stage of the embryos, incubation was started with a 1:1 mixture of AUF and seawater midway through the acclimation process described in Tomita et al [3]. The mixing ratio of AUF to seawater was subsequently adjusted to 1:3, 1:7, and 1:15 during incubation.

The chemical composition of the incubation fluid (sodium, urea, and ammonia concentrations) was measured daily using a DRI-CHEM NX600 automated clinical chemistry analyzer (Fujifilm Co., Tokyo, Japan). Because ammonia concentrations increased over time, the fluid was replaced as necessary to prevent ammonia levels from exceeding 200 µg/dL.

### Basic performance

All 25 embryos were successfully maintained in the system for up to 48 days, until they reached birth size (approximately 16 cm total length). Changes in the chemical composition of Aquarium 1 during the incubation period are shown in Fig. 2B. Sodium concentrations gradually increased while urea concentrations decreased, approaching seawater levels. Due to fluid replacement every 3–4 days, ammonia concentrations were maintained below 200 µg/dL, consistent with previous successful incubations. When the external yolk sac was completely absorbed, embryos were transferred to a 500-L seawater tank maintained at 12 °C for artificial delivery.

### Spatial separation of embryos

**Fig. 3:**
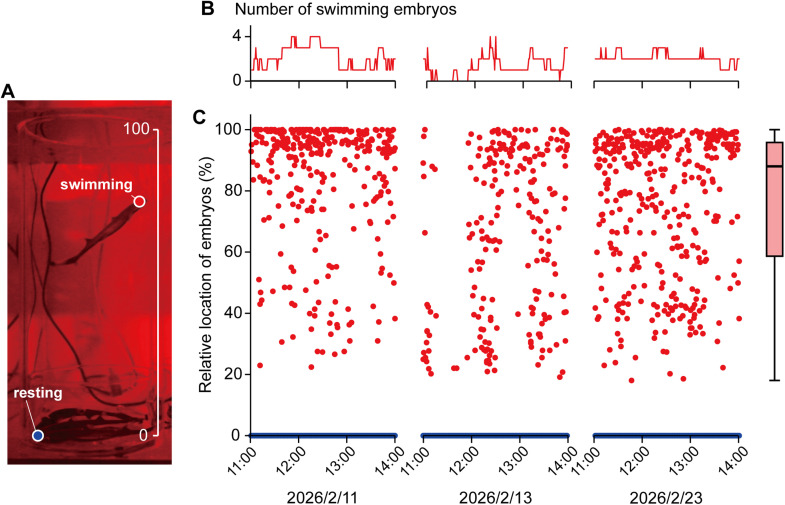
Spatial use of embryos incubated in cylinder-container unit. **A**. Snout positions of resting (blue dot, position = 0 %) and swimming (red dot) embryos were measured. **B**. The number of swimming embryos (out of 12 embryos) during observation period. **C**. Spatial distribution of swimming (red) and resting (blue) embryos during observation period. Box plot represents minimum, lower quartile, median, upper quartile, and maximum values of the swimming embryo positions.

Embryonic activity was recorded using a high-sensitivity video camera (Canon ME20F-SH; Canon Inc., Tokyo, Japan) in Aquarium 1 containing three cylinder–container units. Four embryos were housed per unit. Three-hour recordings (11:00–14:00) were conducted on February 11, 13, and 23, 2026. From these recordings, we sequentially captured one frame per minute using FFmpeg version 8.0.1 (FFmpeg developers), and serial images were created. These images were then imported into ImageJ version 1.54 g (National Institutes of Health, Bethesda, USA), and the snout position of each embryo relative to the aquarium height (aquarium bottom = 0, water surface = 100) was measured using the coordinate measuring tool in ImageJ (Fig. 3A).

Based on the 9-hour observation, an average of 1.83 out of 12 embryos were actively swimming at any given time (range: 0–4; Fig. 3B). Spatial tracking analysis showed that activated embryos mainly occupied the upper third of the acrylic cylinder, spending half of their time at a median height of 88.0 % from the bottom of the aquarium (interquartile range: 58.6–95.8 %; Fig. 3C). This distribution suggests that activated embryos were spatially well separated from resting embryos.

### Oxygen stability

To evaluate oxygen dynamics, dissolved oxygen (DO) was measured hourly in all six incubation units in Aquarium 1 and Aquarium 2 between 12:00 and 18:00 on February 20, 2026, using a portable YSI ProODO sensor (YSI, Inc., Ohio, USA). DO was measured inside each unit and in the surrounding aquarium water. Oxygen consumption within each unit was estimated from the difference between internal and external DO levels.

The effects of embryo number and activity level (proportion of swimming embryos within the incubation unit) on oxygen consumption were analyzed using a linear mixed-effects model (LMM), with embryo number and swimming proportion as fixed effects and incubation unit ID as a random intercept. Analyses were performed using R version 4.5.2 (R Foundation for Statistical Computing) with the *lme4* and *lmerTest* packages.

The mean DO in the cylinder–container units was 95.7 %. DO levels in each unit were highly stable over time, fluctuating within approximately 1 %. During incubation experiments conducted in 2023–2024, embryos exhibited abnormal behavior (absence of response to the light stimuli, lateral body orientation and rapid respiration) when oxygen levels decreased to approximately 70 %, suggesting that this level represents the lower tolerance limit. Thus, DO levels maintained in the new system were within a safe range.

Neither embryo number (β = 3.55 ± 1.71 SE, *p* = 0.107) nor the proportion of swimming embryos (β = −1.21 ± 1.06 SE, *p* = 0.259) significantly affected oxygen consumption within an incubation unit, indicating that oxygen levels remained stable regardless of embryo number or swimming activity.

### Comparison to the previous method

This system is distinguished from previous systems by its ability to safely maintain multiple full-term embryos within the same container. The incubation space per embryo was 3–4 L, approximately one-fifteenth of that required in the original artificial uterine system (50 L; [2]) and half that of the “portable artificial shark uterus” (7 L; [5]).

The new system also has operational advantages in fluid exchange. In the individual-housing system, each incubation container and its fluid must be replaced frequently to maintain a clean environment (see Tomita et al., [5], for protocol details). The total number of operations increases with the number of containers and becomes particularly time-consuming when incubating large numbers of embryos. In the new system, multiple embryos can be maintained within the same container, greatly reducing the number of containers and the time required for fluid exchange.

Despite these advantages, it should be noted that the new system has a higher risk of embryonic mass mortality compared with previous methods. In the earlier systems, embryos were maintained under semi-isolated conditions; thus, mortality of one embryo did not directly affect others. In contrast, in the new system, mortality of a single embryo may directly disrupt the shared environment and potentially induce mass mortality among co-housed embryos. Early detection of embryonic death will therefore be critical, and introduction of higher-resolution chemical (e.g., ammonia concentration) monitoring system should be effective for creating safer system.

## Limitations

This method is only applicable to elasmobranchs (sharks and batoids) with yolk-sac viviparity, characterized by embryonic development that depends solely on the yolk. In contrast, it cannot be applied to species exhibiting other viviparous modes (e.g., lipid histotrophy, oophagy) in which mothers supply additional nutrition to embryos.

Despite the lack of obvious adverse effects on embryonic development, long-term influence after delivery has not yet been evaluated. Extended monitoring of post-delivery outcomes is essential to confirm the long-term safety of this method.

## Related research article

None

## For a published article

None

## Declaration of generative AI and AI-assisted technologies in the manuscript preparation process

During the preparation of this work, the authors used Gemini 3 to revise grammatical errors in the manuscript. After using this tool/service, the authors reviewed and edited the content as needed and take full responsibility for the content of the published article.

## Ethics statements

The handling of the animals was done in strict accordance with the guidelines for animal experiments of the Okinawa Churashima Foundation, with the same consideration for animal care and welfare as that for “higher” vertebrates (reptiles, birds, and mammals). As the guidelines stipulate, the approval from the Institutional Animal Care and Use Committee of Okinawa Churashima Foundation, required for higher vertebrates, is waived for “lower” vertebrates, including fishes.

## CRediT author statement

**Taketeru Tomita**: Conceptualization, Formal analysis, Investigation, Data curation, Writing – original draft. **Atsushi Kaneko**: Conceptualization, Methodology, Investigation, Writing – review & editing. **Tomoya Takeda**: Investigation, Writing – review & editing. **Keiichi Sato**: Supervision, Writing – review & editing.

## Declaration of competing interest

The authors declare that they have no known competing financial interests or personal relationships that could have appeared to influence the work reported in this paper.

## Data Availability

Data will be made available on request.
